# Case report: Primary intracranial EWs/PNET in adults: Clinical experience and literature review

**DOI:** 10.3389/fonc.2022.1035800

**Published:** 2022-10-13

**Authors:** Xianwen Hu, Qi Huang, Ju Wang, Dandan Li, Pan Wang, Jiong Cai

**Affiliations:** ^1^ Department of Nuclear Medicine, Affiliated Hospital of Zunyi Medical University, Zunyi, China; ^2^ Department of Obstetrics, Zunyi Hospital of Traditional Chinese Medicine, Zunyi, China

**Keywords:** Ewing sarcomas, primitive neuroectodermal tumors, EWs/PNET, intracranial, magnetic resonance imaging, PET/CT

## Abstract

**Introduction:**

Adult primary intracranial Ewing sarcomas (EWs)/primitive neuroectodermal tumors (PNETs) are extremely rare, with only 30 patients published before us. The imaging features and treatment strategies of primary intracranial EWs/PNETs are unclear due to its rarity. The aim of this study was to investigate the clinical features, imaging findings, treatment, survival analysis, and prognosis of adult EWs/PNETs, and a systematic review was conducted based on the patient we treated and published literature.

**Case description:**

A 19-year-old male patient suffered from head pain due to an accidental fall on a motorcycle that occurred more than 10 days before going to the hospital, and underwent computed tomography (CT) examination; it was found that the left temporo-occipital fossa was occupied. Magnetic resonance imaging (MRI) was recommended to understand the nature of the lesion, and the result showed that it has a high probability of being a meningioma. He underwent surgical removal of the mass under general anesthesia, and surprisingly, postoperative pathology revealed EWs/PNET. The disease has a high degree of malignancy, and the patient developed multiple metastases throughout the body 5 years after surgery.

**Conclusion:**

Primary intracranial EWs/PNETs in adult patients are rare, of which imaging findings should be considered as one of the differential diagnoses of meningioma, hemangiopericytoma, and malignant triton tumor. Larger solid-cystic masses with septum-like enhancement may be relatively specific imaging findings of intracranial EWs/PNETs. The prognosis of primary adult intracranial EWs/PNETs is poor. Radical tumor resection combined with radiotherapy and chemotherapy is currently the main and possibly the most effective treatment method.

## Introduction

Ewing sarcomas (EWs) and primitive neuroectodermal tumors (PNETs) share the same genetic and histological features; thus, they are collectively referred to as EWs/PNETs, which originate in the neuroectoderm and are mainly composed of primitive neuroectodermal cells (1). EWs/PNETs are highly malignant small round cell tumors with multi-directional differentiation potential, and can be divided into two types according to their origin in bone or soft tissue: intraosseous and extraosseous (2). About 85%–90% of the patients with EWs/PNET show the typical chromosomal translocation t(11;22)(q24;12), which leads to the fusion of the gene EWSR1 and the ETS family gene FLI1 (1, 3). The clinical manifestations of intracranial EWs/PNETs usually include headache, vomiting, dizziness, and other symptoms of intracranial hypertension, and some patients may manifest as eye movement disorders, decreased vision, and unsteady gait (4). Laboratory tests for intracranial EWs/PNETs are usually non-positive, of which imaging studies are often misdiagnosed as meningiomas due to their rarity and mostly dural origin, and the diagnosis of the disease is mainly based on pathology and immunohistochemistry. The prognosis of intracranial EWs/PNETs is poor, associated with age more than 14 years at diagnosis, initial tumor volume more than 200 ml, being male, development of metastases, etc. (2, 5, 6). The primary intracranial EWs/PNETs are extremely rare, the incidence of which is lower in adults than in children, and the published literature is only found in case or case series. Herein, we report a 19-year-old male patient with pathologically confirmed EWs/PNET originating in the left temporo-occipital fossa, who was discovered incidentally on imaging after an accidental fall. Moreover, we reviewed the published literature on adults (≥18 years of age) with intracranial EWs, summarized the radiological and clinical features of this rare tumor, and discussed its imaging differential diagnosis in detail.

## Case description

**Figure 1:**
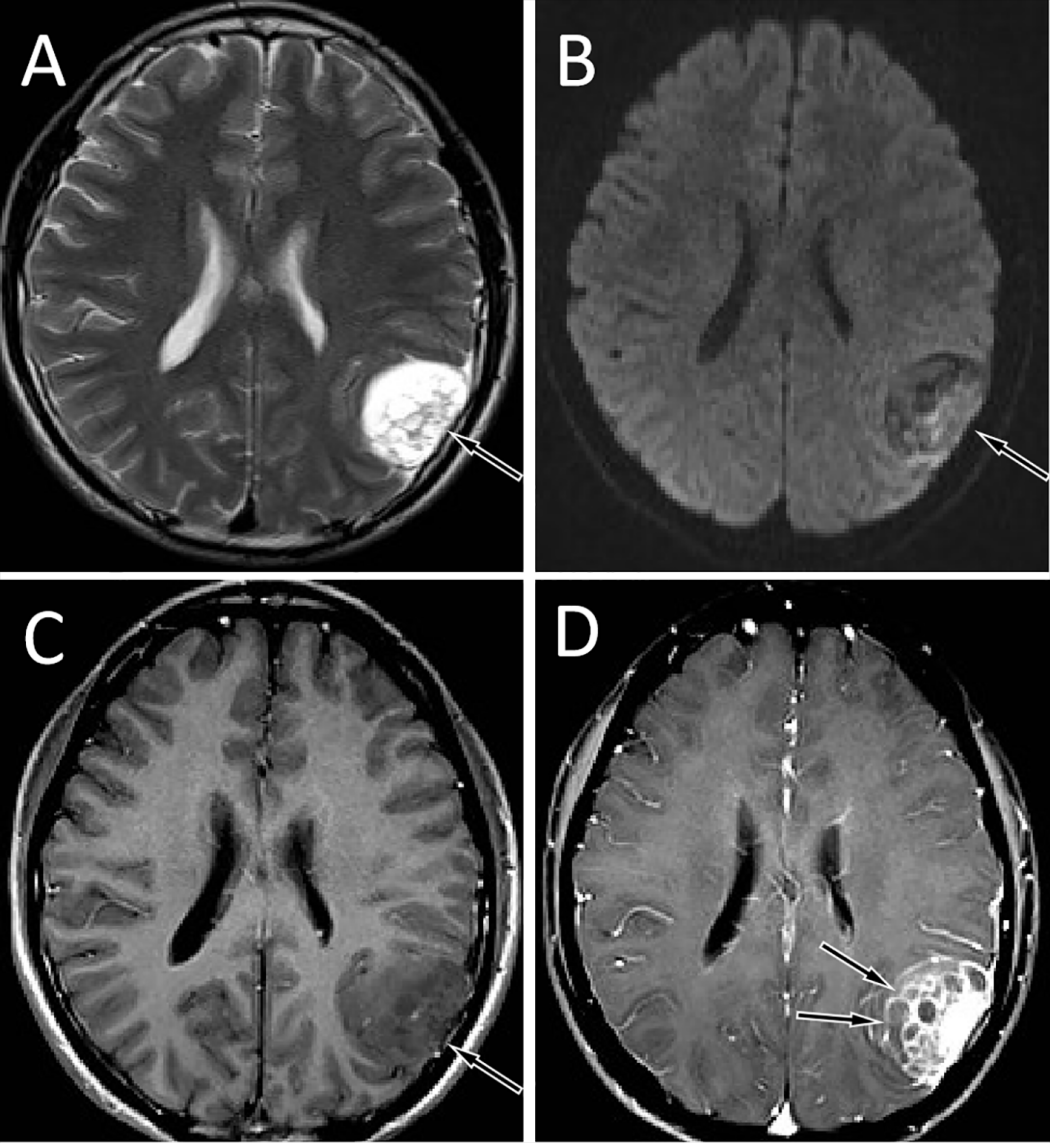
Brain MRI showed that the tumor was located in the left temporo-occipital fossa. T2WI sequence showed that the lesion was high signal (**A**, arrow). Diffusion-weighted imaging showed that the movement of water molecules was not restricted and showed heterogeneous low signal (**B**, arrow). The T1WI sequence showed that the lesion was slightly low signal (**C**, arrow), and the solid component of the tumor was significantly enhanced on contrast-enhanced T1WI, with multiple unenhanced cysts around it, and septum-like enhancement was seen in between (**D,** arrows).

**Figure 2:**
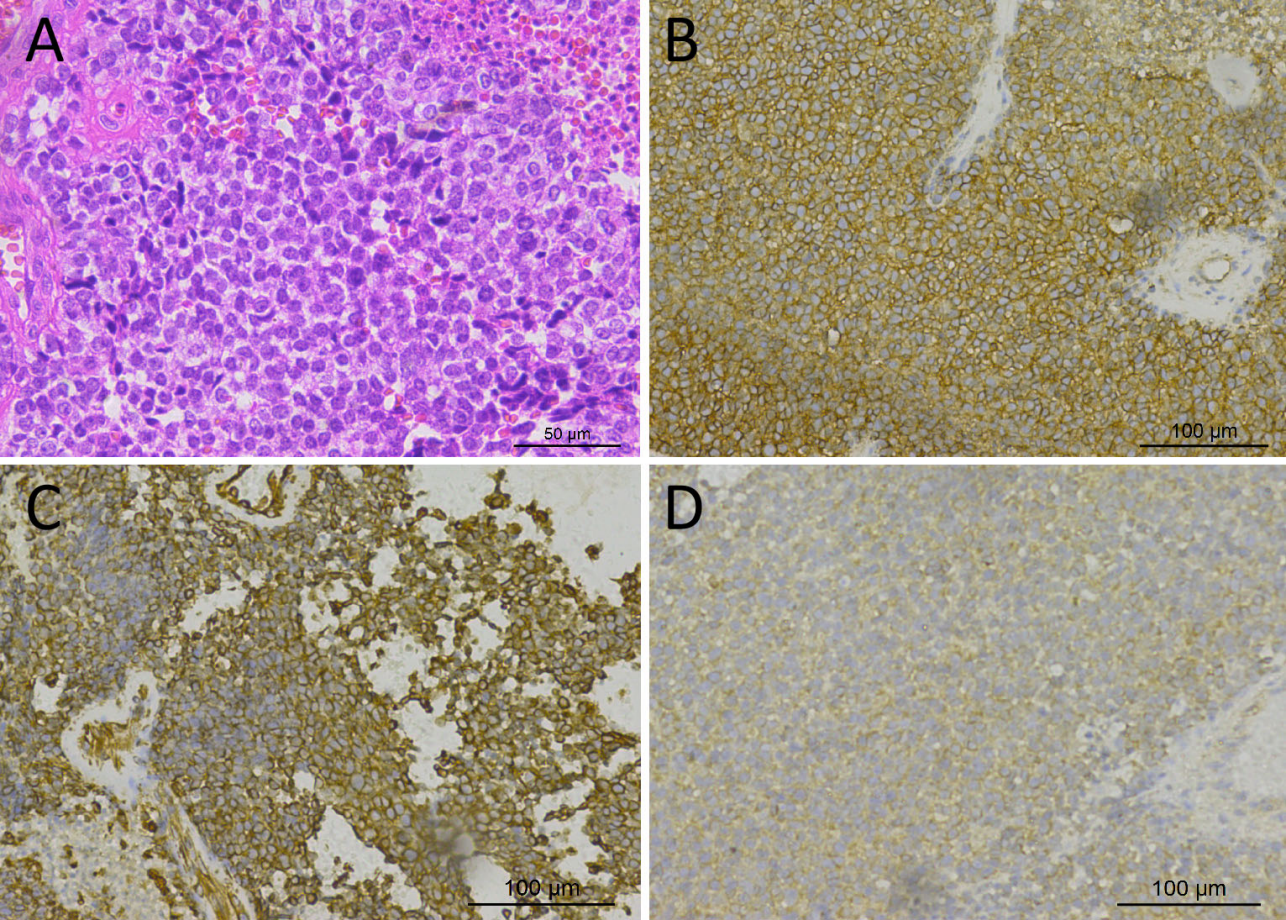
Hematoxylin–eosin staining showed that the tumor tissue was composed of small round cells with relatively uniform morphology, and the nuclei were deeply stained **(A)**. Immunohistochemical staining showed that tumor cells positively expressed CD99 **(B)**, Vimentin **(C)**, and Bcl-2 **(D)**.

**Figure 3:**
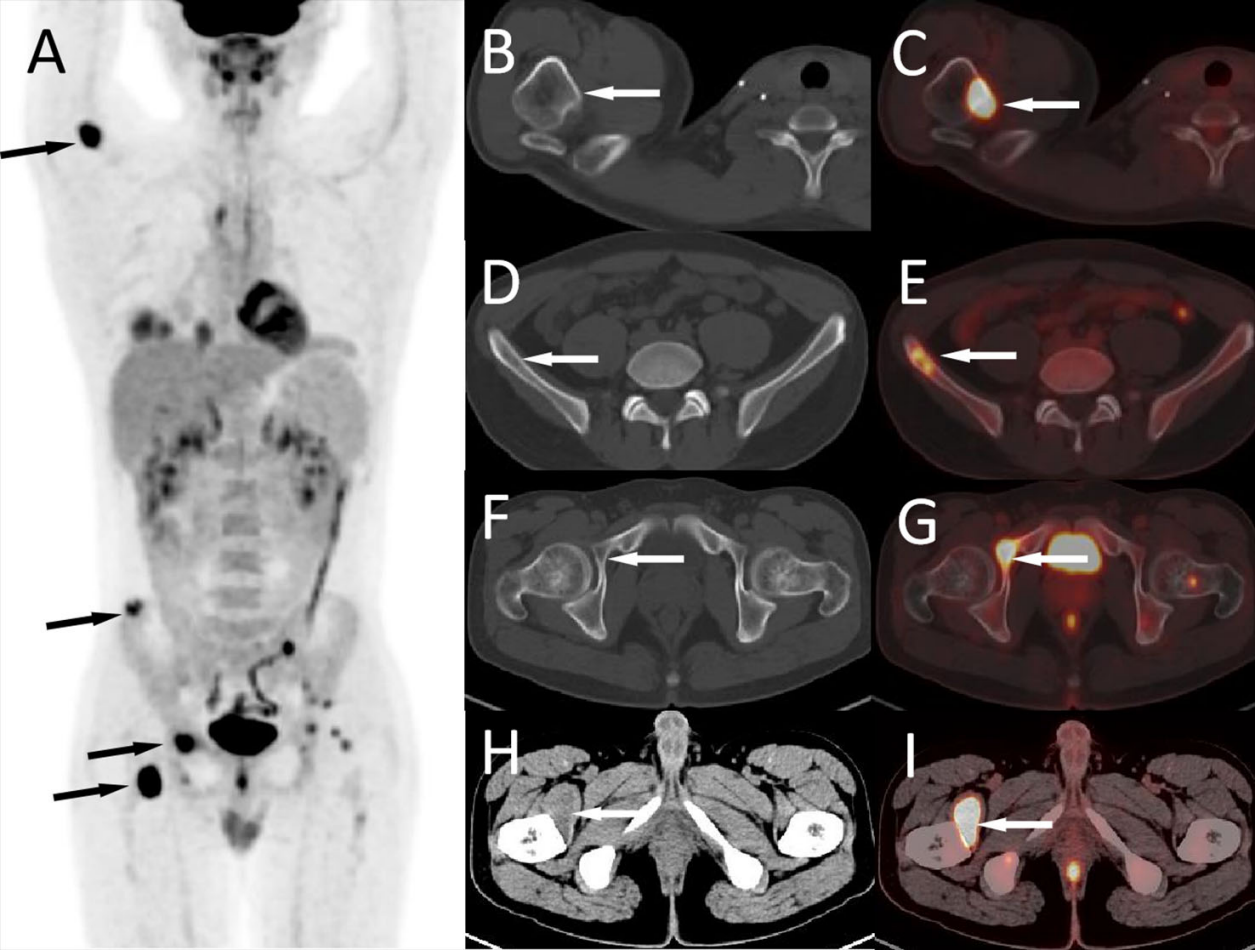
The patient’s ^18^F-FDG PET/CT at 5 years after operation; the maximum intensity projection figure showed multiple FDG concentration foci throughout the body (**A**, arrows). Axial figures showed that the right humerus (**B**, CT; **C**, PET/CT fusion), ilium (**D**, CT; **E**, PET/CT fusion), and acetabulum (**F**, CT; **G**, PET/CT fusion) can be seen with iso-density or slightly hypodense bone destruction areas with high radioactive uptake (arrows). Moreover, a hypodense mass with FDG accumulation was seen in the right iliopsoas muscle (**H**, CT; **I**, PET/CT fusion, arrows).

A 19-year-old male patient suffered from head pain due to an accidental fall on a motorcycle that occurred more than 10 days before going to the hospital, and underwent computed tomography (CT) examination; a mass was found in the left temporo-occipital fossa. He was admitted to our hospital for further diagnosis and treatment. His parents were healthy and no family members had a history of similar diseases. Magnetic resonance imaging (MRI) was recommended to understand the nature of the lesion, and the result showed that it has a high probability of being a meningioma (as shown in **Figure 1**). No obvious positive signs were found in physical examination, and all laboratory indexes were within the normal reference value range. Based on the established diagnosis of a left temporo-occipital fossa space-occupying lesion, the patient underwent surgical resection of the tumor under general anesthesia. During the operation, the left temporoparietal horseshoe-shaped skin flap was taken, and the scalp was incised in full thickness to expose the skull and then a bone flap with a size of about 6 cm × 6 cm was removed. After the bone flap was taken out, it was found that the dura of about 2 cm × 2 cm was invaded by the tumor and broke through, and the inner plate of the covering skull was not invaded by the tumor. The dura was cut along the edge of the tumor invading the dura, and the tumor was removed together. The tumor tissue was gray, with clear demarcation from the surrounding brain tissue, abundant blood supply, soft texture, and a size of about 4.0 cm × 5.0 cm × 4.0 cm. Postoperatively, the resected tumor tissue was sent for pathological examination, and the results showed that there were diffusely distributed round cells in the tumor (as shown in **Figure 2**). Immunohistochemical staining showed that tumor cells positively expressed CD99, Vimentin, Bcl-2, ki-67 (about 40%), weakly expressed CD56, SMA, and negatively expressed CD34, CgA, CK, Desmin, EMA, S100, etc. Ewing’s sarcoma breakpoint region 1 (EWSR1) gene rearrangement was identified by fluorescence *in situ* hybridization in a foreign hospital. Based on these findings, the patient was diagnosed with intracranial extraosseous EWs/PNET. He did not undergo further radiotherapy and chemotherapy after the operation. At the 5th year after the operation, the patient was re-admitted to the hospital because of multiple bone pains throughout the body. ^18^F-FDG PET/CT was recommended to evaluate the patient’s systemic condition, and the results revealed multiple high radioactive uptake foci throughout the body (as shown in **Figure 3**). Biopsy of the lesion at the iliac bone revealed the same disease as he had previously suffered. He then received a vincristine–cyclophosphamide–doxorubicin chemotherapy regimen and palliative radiation targeting the lesions of the humerus, ilium, and acetabulum. Unfortunately, he passed away 3 months after starting maintenance therapy with chemoradiotherapy.

## Literature review

Primary intracranial EWs/PNET case reports and case series published in PubMed, Embase, and Web of Science databases as of 1 June 2022 were searched, with language restrictions limited to English. The following keywords were used: Ewing’s sarcoma, EWs, primitive neuroectodermal tumors, PNET, intracranial, and dura mater. The first author, publication year and country, as well as the patient’s age, gender, main clinical symptoms, CT and MRI imaging findings, treatment methods, and follow-up results were recorded for each case (as shown in **Table 1**).

**Table 1:** Clinical and imaging features of the cases of primary intracranial EWs/PNETs.

Author, year, country	Gender/age	Main symptoms and signs	Onset of symptoms (months)	Location	Morphological	CT^a^	MRI		CECT/MRI Degree/septum-like	MD (cm)	Metastases at diagnosis	Treatment	Recurrence	Metastasis	Follow-up/(months)
							T1WI	T2WI							
Batur/2021/Turkey (7)	44/M	Headache	4	Left posterior fossa	Solid-cystic	–	Slightly high	Isointense	Obvious/Y	–	N	Surgery	N	N	Alive/36
Deshpande/2021/India (8)	33/M	Headache, nausea, vomiting	–	Left temporal fossa	Solid-cystic	–	–	–	Obvious /Y	–	N	Surgery, focal RT (55.8 Gy/31#), COG protocol (IE/VAC, 48 weeks)	N	N	Alive/18
Deshpande/2021/India (8)	33/F	Left eye movement impairment	–	Left frontal fossa	–	–	–	–	–	–	N	Surgery, focal RT (54 Gy/30#), vincristine + cisplatin (2 weeks), discontinued in view of intolerance	Y	Vertebral	Alive/17
Jiang/2020/China (4)	55/F	Memory decline	1	Left frontal fossa	Solid-cystic	Slightly higher density	Hypointense-to-isointense	Hypointense-to-hyperintense	Obvious/Y	6.5	N	Surgery, focal RT (55 Gy/30#)	N	N	Alive/18
Howell/2020/USA (9)	37/M	Dizziness, headache, nausea, vomiting	1	Multifocal (pineal region, third and fourth ventricles)	Solid-cystic	–	–	–	–	–	Y (Spinal cord)	4 weeks vincristine, 36 Gy	N	N	Alive/7
Huang/2020/Australia (10)	19/M	Headache, vomiting	0.5	Left frontal fossa	Solid-cystic	High density	Hypointense-to-isointense	Hypointense-to-hyperintense	Obvious/Y	7.1	N	Surgery, VIDE+VAI, focal RT	N	N	Alive/12
Chen/2019/China (11)	23/M	Swelling over the scalp, headache	2	Left temporoparietal fossa	Solid-cystic	–	Hypointense-to-isointense	Hypointense-to-hyperintense	Obvious/Y	–	Bone involvement	Surgery, 55 Gy	N	Spinal cord	Died/6
Chen/2019/China (11)	18/M	Headache, swelling over the scalp	7	Left temporal fossa	Solid-cystic	–	Hypointense-to-isointense	Hypointense-to-hyperintense	Obvious/Y	–	Bone involvement	Surgery, 50 Gy	Y	N	Died/23
Chen/2019/China (11)	43/M	Epilepsy	1	Right parietal fossa	Solid-cystic	–	Hypointense-to-isointense	Hypointense-to-hyperintense	Obvious/Y	–	N	Surgery, VAC+ actinomycin D, 50 Gy	Y	N	Died/48
Chen/2019/China (11)	22/F	Swelling over the scalp	1	Right temporal fossa	Solid-cystic	–	Hypointense-to-isointense	Hypointense-to-hyperintense	Obvious/Y	–	Bone involvement	Surgery, VAC, 55 Gy	Y	Skull	Died/38
Ke/2017/China (12)	43/M	Epilepsy	–	Right parietal fossa	–	–	Hypointense-to-isointense	Hypointense-to-hyperintense	Obvious/Y	–	N	Surgery, VDAC	Y	N	Died/48
AndenHeuvel/2015/USA (13)	61/M	Left hemiparesis	2	Right frontal and temporal fossa	Solid-cystic	–	–	–	Ring reinforcement	6.2	N	Surgery	N	N	–
Salunke/2014/India (14)	52/M	Fever, headache	1	Posterior fossa	Solid-cystic	–	–	–	Obvious/Y	6.0	Bone involvement	Surgery, VAC + ifosfamide, focal RT 50 Gy	Y	N	Died/6.5
Cole/2014 /USA (15)	51/F	Visual disturbances	–	Posterior fossa	Solid-cystic	–	–	–	Obvious/Y	3.53	N	Surgery, VCD (alternated with EI every 3 weeks) for 14 cycles. After the 4th cycle, doxorubicin was displaced into dactinomycin	N	N	Alive/24
Huguenard/2021/USA (16)	34/M	Headache, back pain	3	Right frontal fossa	Solid-cystic	High density	Hypointense-to-isointense	Hypointense-to-hyperintense	Obvious/Y	3.7	Pia mater, meninges	Surgery, VC + cisplatin, FOCAL RT	Y	Spinal cord	Died/6
Mellai/2010/Italy (17)	56/F	Headache, confusion	–	Right temporal fossa	Solid-cystic	–	–	–	Obvious/Y	–	N	Surgery	N	N	Alive/18
Attabib/2006/Canada (18)	48/F	Headache, left ptosis	6	Left cavernous fossa	–	–	–	–	Obvious/N	4.0	N	Surgery, VCD (alternated with EI every 3 weeks), 54 Gy	N	N	Alive/14
D'Antonio/2004/Italy (19)	50/F	Headaches, vomiting	–	Right parieto- temporal fossa	–	–	–	–	Obvious/Y	6.0	N	Surgery	N	N	Alive/12
Simmons/2001/UK (20)	67/F	Headache, facial paralysis	18	Right CA	–	–	–	–	–	–	Bone involvement	Palliative radiation	Y	Meninges	Died/13
Kalamarides/2001/France (21)	34/F	Vertigo, tinnitus	12	Left CA	Solid	–	–	Hyperintense	Obvious/N	1.8	N	Surgery, craniospinal radiotherapy (35 Gy with an overdosage to 55 Gy over the tumor bed)	Y	N	Alive/12
Kalamarides/2001/France (21)	41/F	Dizziness, left deafness	12	Left CA	Solid	–	–	Hyperintense	Obvious /N	1.4	N	Surgery, craniospinal radiotherapy (35 Gy with an overdosage to 55 Gy over the tumor bed)	N	N	Alive/12
Tanboon/2012/Canada (22)	22/F	Headache, vomiting, blurred vision	3	Right frontal fossa	Solid-cystic	–	–	–	Obvious/Y	4.0	Bone involvement	Surgery	Y	Multiple bones, cervical LNs	Died/13
Antonelli/2011/Italy (23)	37/M	Headache, coma	0.5	Right frontotemporal fossa	Solid-cystic	Isodensity	–	–	Obvious/Y	–	N	Surgery, RT 6 weeks	Y	N	Alive/17
Mobley/2006/USA (24)	21/M	Headache, double vision	2	Right occipital fossa	Predominantly solid	Isodensity	–	–	Obvious/Y	3.5	N	Surgery, Dactinomycin, VAC, 54 Gy	Y	Cervical and thoracic spine	Alive/17
Idrees M/2005/ USA (25)	46/M	Headache, nausea, and vomiting	0.5	Cavernous sinus and sella	Solid	Isodensity	Isointense	Hyperintense	Obvious/N	2.3	N	Vincristine, 5,400 cGy	–	–	–
Ishii/2001/Japan (26)	19/F	Headache	–	Right frontal fossa	–	–	–	–	–	–	N	Surgery, VAC, palliative radiation	Y	–	Died/36
Papotti/1998/Italy (27)	30/F	Headache, dizziness	–	Right frontal fossa	Solid-cystic	–	–	–	–	7.0	N	Surgery, VAC, 50 Gy	Y	Thoracic spine	Died/120
Haveman/2020/Netherlands (28)	53.76/M	–	–	Temporal fossa	–	–	–	–	–	–	LNs, CNS	Surgery, 6xVIDE, 7xVAC, RT	–	–	Died/15
Haveman/2020/Netherlands (28)	30.36/F	–	–	Frontal fossa	–	–	–	–	–	–	Bone involvement	Surgery, 6x VIDE, 8x VAI, RT	–	–	Died/38
Haveman/2020/Netherlands (28)	69.34/M	–	–	Parieto-occipital	–	–	–	–	–	–	N	Surgery, 6x VIDE, 7x VAC, RT	–	–	Died/15
Current case	19/M	Accidental discovery	N	Left temporal fossa	Solid-cystic	Isodensity	Slight hypointense	Hyperintense	Obvious/Y	5.0	N	Surgery, VAC, palliative radiation	N	Multiple bones and muscles	Died/66

a refers to the solid component density of the tumor. M, male; F, female; Y, yes; N, not; CT, computed tomography; CNS, central nervous system; MRI, magnetic resonance imaging; CE, contrast enhancement; CA, cerebellopontine angle; R, radiotherapy; LNs, lymph nodes; VAC, vincristine, doxorubicin, cyclophosphamide; VCE, vincristine, cyclophosphamide, epirubicin; VIDE + C, vincristine, ifosfamide, doxorubicin, etoposide plus cyclophosphamide; VIDE, vincristine, ifosfamide, doxorubicin, etoposide; VTI, vincristine, temozolomide, irinotecan; VAI, vincristine, actinomycin D; RT, radiotherapy.

**Figure 4:**
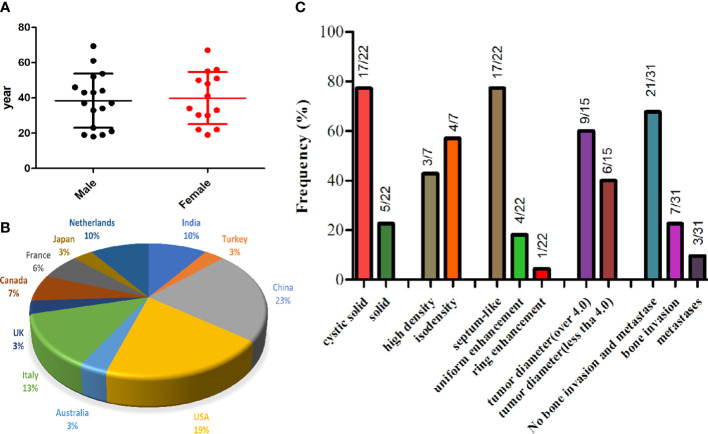
The gender and age distribution **(A)** and country proportion **(B)** of 31 adult patients with primary intracranial EWs/PNET, and imaging features of primary intracranial EWs/PNET **(C)**.

**Figure 5:**
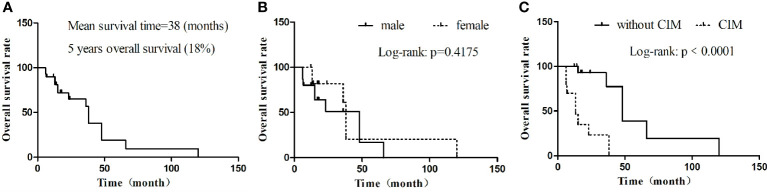
Kaplan–Meier OS. **(A)** OS in all cases; **(B)** OS by gender; **(C)** OS by whether there are cranial invasion or distant metastasis. CIM, cranial invasion or distant metastasis; MST, median survival time; OS, overall survival.

Through a systematic search, 30 adult primary intracranial EWs/PNETs were published prior to our present case (1, 4, 7–28). A total of 31 patients including our patient, consisted of 17 male and 14 female patients with a median age of 37 years, with the top three cases published from China (23%), USA (19%), and Italy (13%), and the detailed distribution of patients is shown in **Figures 4A, B**. Most patients came to the hospital for medical assistance due to short-term headache, dizziness, vomiting, and visual disturbances. Rare clinical symptoms include epilepsy, fever, memory loss, skull invasion in some patients, and local signs of scalp soft tissue swelling. The main foci originated from frontal fossa (7/31), temporal fossa (5/31), followed by posterior fossa, parietal fossa, pontine cerebellar triangle, frontotemporal fossa, frontoparietal fossa, parietal fossa junction, cavernous sinus, etc. Most of the tumor foci were solid-cystic; only four cases were solid with small diameters, ranging from 1.4 to 3.5 cm. The solid component of the tumor showed isodense or slightly high density on CT, slightly low or isointense on T1WI, and hypointense-to-hyperintense on T2WI. On contrast-enhanced CT or T1WI, most of the solid components of the tumor were significantly enhanced, and septum-like enhancement was seen in the cystic components; only one patient showed ring enhancement. Solid tumors all showed obvious uniform enhancement. Of all adults with primary intracranial EWs/PNETs, seven had cranial involvement at diagnosis, and three had meningeal or spinal metastases (as shown in **Figure 4C**). Most of the patients received surgical resection of the tumor combined with radiotherapy and chemotherapy. Overall, intracranial EWs/PNET has a poor prognosis, with a median survival of 38 months and a 5-year survival rate of only 18%. There was no significant difference in overall survival between male and female patients, and cranial invasion or distant metastasis at the first visit was a prognostic factor (as shown in **Figure 5**).

## Discussion

Intracranial EWs/PNETs are rare and account for only 0.03% of all intracranial tumors (29), which is more common in children and adolescents, and previous studies have shown that the median age at diagnosis of intracranial EWs/PNET is 15 years, and there are slightly more male than female patients (30). The case reported here is an intracranial EWs/PNET in the left temporo-occipital fossa of a 19-year-old male patient who was found incidentally after a trauma and had no previous clinical symptoms. In addition, we reviewed all cases of primary intracranial EWs/PNET in adults (≥18 years of age); 30 patients had been reported before our case. The oldest reported patient with primary intracranial EWs/PNET was 69 years old, and the median age at diagnosis of adult patients was 37 years. Our current study shows that the supratentorial fossa including the temporal fossa and frontal fossa are the most common sites for intracranial EWs/PNET, and the most common clinical manifestations include headache, dizziness, vomiting, and visual disturbances, without specificity.

Imaging tests including CT and MRI play a significant role in the diagnosis of central nervous system diseases, especially MRI. On imaging, primary intracranial EWs/PNET is mostly a mass that grows with the dura mater as a wide base, which can grow across the cranial fossa and midline of the brain. Large cystic necrotic areas can be seen in most tumors, and hemorrhage can be seen in some patients, but few calcifications appear. The adjacent skull may be compressed and thinned or invaded to cause bone destruction. Our study showed that intracranial EWs/PNETs were mostly solid-cystic masses, in which the solid components were mostly isodense or slightly hypodense on CT, and only a few small masses showed uniform density. On the T1WI sequence of MRI, the solid component of the tumor was slightly hypointense or isointense, and T2WI was hypo-to-hyperintense, while the cystic part was hypointense on T1WI sequence and hyperintense on T2WI. On contrast-enhanced CT or T1WI, the solid part of the tumor in most patients was significantly enhanced, and the adjacent dura mater was significantly enhanced; only a patient showed primary ring-enhancing cystic lesions in all published cases (13). Notably, on contrast-enhanced scans, septum-like enhancement was seen in cystic regions of tumors in our patient and most of the published literature, which we believe is different from other tumors. Intracranial EWs/PNET including our patient and the published cases were misdiagnosed as meningioma or hemangiopericytoma (HPC) preoperatively; thus, these two tumors are the most important imaging differential diagnoses for intracranial EWs/PNET. Meningiomas are common adult central nervous system tumors, mostly benign. Meningiomas on CT are mostly isodense or high-density spherical or hemispherical masses, with a higher probability of calcification, but with less low-density cystic necrosis, and even if present, the volume of cystic necrosis is small (31). On MRI, the signal of meningiomas is relatively uniform, and the T1WI and T2WI sequences are iso-to-hyperintense, and hyperintensity is more common on DWI, while our patient was hypointense on DWI, which is different from it. Angiography can clearly show the internal and external carotid arteries supplying blood to the meningioma. Typical signs include the tumor body compressing adjacent blood vessels, but the tumor body and its interface are still smooth, showing a “holding ball” change (32). HPC is a malignant tumor with abundant blood vessels, which often leads to low-density necrotic foci due to insufficient local blood supply to the tumor, and the morphology of the tumor is irregular, most of which is lobulated (33), while the shape of EWs/PNET is more regular. On contrast-enhanced scans, HPC was more enhanced and intratumoral vascular flow void signals were more common, and angiography showed multiple tortuous and thickened vascular shadows in the mass (34), which is different from EWs/PNET. Moreover, malignant triton tumor (MTT), namely, malignant schwannoma with rhabdomyoma differentiation, another rare intracranial tumor, is often misdiagnosed as meningioma in previous literature reports; thus, it should also be considered as one of the differential diagnoses of EWs/PNET. MTT is usually equal and slightly hyperdense on CT, and a small volume of cystic necrosis is often seen. Tumors on MRI are mostly low signal on T1WI and high signal on T2WI, and arc or linear low-signal shadows appear in the high-signal tumor tissue, which is relatively specific for the diagnosis of MTT (35).

The diagnosis of EWs/PNET mainly depends on pathology and immunohistochemistry. Under the microscope, the tumors are composed of small round cells or elliptic cells, with hyperchromatic nuclei, and mildly basophilic cytoplasm and Homer–Wright chrysanthemum or Flexner–Weinsteiner chrysanthemum structures can be seen in some tumors (2, 29). Immunohistochemistry showed that almost all tumor cells of intracranial EWs/PNET patients consistently and diffusely expressed CD99, but there was no specificity (36). In addition, tumor cells expressed Vimentin, NSE, Syn, and membrane protein FLI-1 to varying degrees, and molecular detection to find EWSR1 gene rearrangement is the current gold standard for diagnosing EWs/PNETs (2, 12).

Due to its rarity, the standard treatment regimen for intracranial EWs/PNET has not been established, and surgery for the purpose of total tumor resection is currently the main treatment method. In our review of the published literature, most patients were treated with a combination of radiotherapy and chemotherapy including vincristine, cyclophosphamide, doxorubicin, ifosfamide, etoposide, doxorubicin, and radiomycin D and other drugs after surgical resection (30). Our study including 31 adults with intracranial EWs/PNET showed that the prognosis was poor, and the longest follow-up was 10 years in a patient who eventually metastasized to the thoracic spine, progressed, and died (27). Among these patients, their median survival was only 38 months, and the 5-year survival rate was less than 20%.

In conclusion, adult patients with primary intracranial EWs/PNET are rare, whose imaging findings should be considered as one of the differential diagnoses of meningioma, HPC, and MTT. There are large cystic lesions in the tumor, and septum-like enhancement on contrast-enhanced scan may be a relatively specific feature of EWs/PNET, whereas the rarity of the disease limits the clinical application of our findings, and more cases and long-term follow-up studies are needed in the future to fully understand EWs/PNET in the adult population.

## Data availability statement

The original contributions presented in the study are included in the article/Supplementary Material. Further inquiries can be directed to the corresponding authors.

## Ethics statement

Written informed consent was obtained from the individual(s), and minor(s)’ legal guardian/next of kin, for the publication of any potentially identifiable images or data included in this article.

## Author contributions

JC and PW: funding acquisition; DL: investigation; QH and JW: methodology; XH: writing—original draft; XH, PW, and JC: writing—review and editing. All authors contributed to the article and approved the submitted version.

## Funding

This study was funded by the National Natural Science Foundation of the People’s Republic of China, NSFC (grant numbers: 81571712), Zunyi Medical College Research Start Fund 2018ZYFY03, and QianKeHe platform talents [2017] (Grant No. 5733-035).

## Conflict of interest

The authors declare that the research was conducted in the absence of any commercial or financial relationships that could be construed as a potential conflict of interest.

## Publisher’s note

All claims expressed in this article are solely those of the authors and do not necessarily represent those of their affiliated organizations, or those of the publisher, the editors and the reviewers. Any product that may be evaluated in this article, or claim that may be made by its manufacturer, is not guaranteed or endorsed by the publisher.
